# Deep roots mitigate drought impacts on tropical trees despite limited quantitative contribution to transpiration

**DOI:** 10.1016/j.scitotenv.2023.164763

**Published:** 2023-10-01

**Authors:** Kathrin Kühnhammer, Joost van Haren, Angelika Kübert, Kinzie Bailey, Maren Dubbert, Jia Hu, S. Nemiah Ladd, Laura K. Meredith, Christiane Werner, Matthias Beyer

**Affiliations:** a IGOE, Environmental Geochemistry, TU Braunschweig, Langer Kamp 19c, 38106 Braunschweig, Germany; b Ecosystem Physiology, University of Freiburg, Georges-Köhler-Allee 53/54, 79110 Freiburg, Germany; c Biosphere 2, University of Arizona, 32540 S Biosphere Road, Oracle, AZ 85623, USA; d Honors College, University of Arizona, 1101 E. Mabel St., Tucson, AZ 85719, USA; e Institute for Atmospheric and Earth System Research, University of Helsinki, P.O. Box 68, Pietari Kalmin katu 5, 00014 Helsinki, Finland; f School of Natural Resources and the Environment, University of Arizona, 1064 E Lowell St, Tucson, AZ 85721, USA; g Isotope Biogeochemistry and Gasfluxes, ZALF, Eberswalder Straße 84, 15374 Müncheberg, Germany; h Department of Environmental Sciences, University of Basel, Bernoullistrasse 32, 4056 Basel, Switzerland

**Keywords:** Biosphere 2, Drought resistance, Plant-water relations, Root water uptake depth, Water content, Water stable isotopes

## Abstract

Deep rooting is considered a central drought-mitigation trait with vast impact on ecosystem water cycling. Despite its importance, little is known about the overall quantitative water use via deep roots and dynamic shifts of water uptake depths with changing ambient conditions. Knowledge is especially sparse for tropical trees. Therefore, we conducted a drought, deep soil water labeling and re-wetting experiment at Biosphere 2 Tropical Rainforest. We used in situ methods to determine water stable isotope values in soil and tree water in high temporal resolution. Complemented by soil and stem water content and sap flow measurements we determined percentages and quantities of deep-water in total root water uptake dynamics of different tree species. All canopy trees had access to deep-water (max. uptake depth 3.3 m), with contributions to transpiration ranging between 21 % and 90 % during drought, when surface soil water availability was limited. Our results suggest that deep soil is an essential water source for tropical trees that delays potentially detrimental drops in plant water potentials and stem water content when surface soil water is limited and could hence mitigate the impacts of increasing drought occurrence and intensity as a consequence of climate change. Quantitatively, however, the amount of deep-water uptake was low due to the trees' reduction of sap flow during drought. Total water uptake largely followed surface soil water availability and trees switched back their uptake depth dynamically, from deep to shallow soils, following rainfall. Total transpiration fluxes were hence largely driven by precipitation input.

## Highlights

• All studied tropical canopy trees accessed water at soil depths up to 3.3 m
• Maximum deep water contribution to transpiration ranged between 21 and 90 % during drought
• Temporal dynamics of deep water uptake shares and amounts differed among individual trees
• Deep roots help tropical trees endure droughts but total transpiration fluxes are driven by precipitation.

## Graphical abstract

**Figure:**
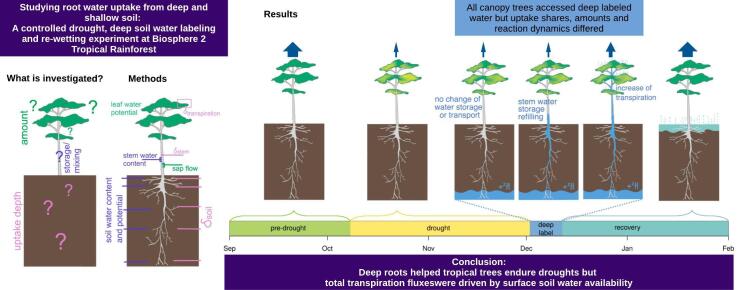

## Abbreviations

Trees: CF
  *Clitoria fairchildiana* R.A. Howard
  CP
  *Ceiba pentandra* L.
  HC
  *Hura crepitans* L.
  HT
  *Hibiscus tiliaceus* L.
  PA
  *Pachira aquatica* Aubl.
DBH: diameter at breast height
RH: air relative humidity
RWU: root water uptake
sd: standard deviation
T_air_: air temperature
TRF: Tropical Rainforest (biome at Biosphere 2)
V_h_: tree sap flux density
VWC_soil_: soil volumetric water content
VWC_stem_: tree stem volumetric water content
WVMR: water vapor mixing ratio
Ψ_md_: midday leaf water potential
Ψ_pd_: predawn leaf water potential
Ψ_soil_: soil matric potential
δ: hydrogen and oxygen stable isotope values
δ^2^H: hydrogen stable isotope value (ratio between ^1^H and ^2^H) in reference to VSMOW standard
δ^18^O: oxygen stable isotope value (ratio between ^16^O and ^18^O) in reference to VSMOW standard

## 1 Introduction

Tropical forests greatly impact terrestrial water (Schlesinger and Jasechko, 2014) and carbon cycling (Gentine et al., 2019). Although humid tropics are characterized by high water availability, precipitation is often seasonal and numerous non-cyclical droughts have been recorded in the past decades (Bonal et al., 2016). In 2015–2016, a severe drought anomaly markedly decreased carbon sequestration and increased tree mortality across the tropics, with relatively slow recoveries in humid tropical forests (Wigneron et al., 2020). This record-breaking drought strongly reduced forest transpiration due to soil water deficit (Meng et al., 2022), stomatal closure and xylem embolism (Fontes et al., 2018). As a consequence of climate change, drought risk is projected to increase in tropical forests (Corlett, 2016). However, despite the global importance of trees in the humid tropics, their drought responses and thus further feedback on climate remain uncertain.

Deep roots, i.e., roots in soil depths ≥1 m (Maeght et al., 2013), play a central role in tree drought tolerance. These roots specialize in taking up water (Germon et al., 2020; Wang et al., 2022) and often compensate when surface soil water is limited (Pierret et al., 2016). Deep soils provide a reliable water source since water availability becomes increasingly decoupled from precipitation events and temporal fluctuations decrease with greater soil depths (Chitra-Tarak et al., 2018). Additionally, deep-water is less accessed by understory vegetation and is protected from soil evaporation. Tropical humid forests mostly have superficial nutrient cycling (Poszwa et al., 2002) and rooting depths, and deep root share has been shown to increase with seasonal water limitation (Schenk and Jackson, 2002; Tumber-Dávila et al., 2022). Nonetheless, the occurrence of deep roots is widespread across species and biomes, while their precise occurrence and significance is still hard to estimate due to sampling bias towards shallow roots (Pierret et al., 2016), which are easier and cheaper to access (Maeght et al., 2013). Uncertainty is especially high for low latitudes due to insufficient sampling schemes across the tropics (Schenk and Jackson, 2002) that rarely exceed 1 m (Pierret et al., 2016) and a general lack of records in moist tropical forests (Schenk and Jackson, 2005). Given the importance of deep roots in modeling tropical ecosystem evapotranspiration (Sakschewski et al., 2021) and a likely underestimation of rooting depth in terrestrial-biosphere models (Tumber-Dávila et al., 2022), more research is urgently needed.

The presence of roots can be determined with excavations (e.g., Niiyama et al., 2010) but data are generally restricted to one point in time and do not quantify root water uptake (RWU). For tropical forests, temporal changes of deep RWU were previously estimated from changes in soil water content (Bruno et al., 2006; Markewitz et al., 2010; Davidson et al., 2011; Christina et al., 2017). However, this requires modeling and RWU cannot be clearly separated from soil water movement nor between different plant individuals. Water stable isotopes are an established tool to investigate RWU of individual plants across soil depth. The isotopic method is based on distinguishable water isotopic compositions of different water sources, e.g., from different soil depths, and the assumption that water within trees reflects the mixture of RWU (Wershaw et al., 1966; Zimmermann et al., 1967; Rothfuss and Javaux, 2017; Amin et al., 2021). By comparing plant water stable isotope values to those of potential water sources, researchers have determined uptake percentages using simple mixing equations, statistical methods and modeling (Rothfuss and Javaux, 2017). This has greatly advanced our understanding of trees' contribution to tropical ecosystem water cycling, including the interplay of seasonal dynamics of RWU depth and niche segregation (Schwendenmann et al., 2015; Sohel et al., 2021), the occurrence of hydraulic lift (Jackson et al., 1999) and the dependency on incoming precipitation (Borma et al., 2022).

The precise location and quantification of deep RWU with water stable isotopes is however hampered by insufficient sampling depth (Beyer and Penna, 2021) and less variable soil water isotopic compositions with depth (Sprenger et al., 2016). Isotopic labeling can substantially lower measurement uncertainty (Rothfuss and Javaux, 2017). However, it is nearly impossible to homogeneously label deep soil water in the field. Additionally, stable isotope studies investigating RWU have traditionally relied on destructive sampling, which only represents discrete points in time (Beyer and Penna, 2021) and thus misses dynamic shifts in RWU arising from changes in stomatal control, atmospheric demand and water availability across soil depths (Rothfuss and Javaux, 2017). Isotope studies specifically investigating RWU of tropical trees at soil depth >1 m are so far limited to one (Brum et al., 2019; Sohel et al., 2021) or few sampling time points surrounding a labeling event (Stahl et al., 2013) or focused on differences between wet and dry season (Romero-Saltos et al., 2005; Borma et al., 2022). Additionally, experiments were only conducted in ecosystems with pronounced seasonality (dry period >3 months) and to the authors' best knowledge, there is only one study from outside the Amazon (Sohel et al., 2021).

Since the invention of field-deployable laser-based analyzers (Gupta et al., 2009), new in situ methods have been developed and are increasingly applied to measure water stable isotopes in soils (Rothfuss et al., 2013; Volkmann and Weiler, 2014; Oerter and Bowen, 2017; Kübert et al., 2020), tree xylem (Volkmann et al., 2016; Marshall et al., 2020; Kühnhammer et al., 2022) and plant transpiration (Simonin et al., 2013; Kübert et al., 2023). This greatly improves our ability to capture temporal dynamics of isotopic fingerprints in ecosystem water fluxes and is a promising avenue for investigating water travel times across the soil-plant-atmosphere continuum (Sprenger et al., 2019) and improving isotope-enabled ecohydrological models (Beyer et al., 2020).

Trees are not simple straws but complex systems with a variety of water flow paths and pools (Evaristo et al., 2019). The high temporal resolution of in situ isotope data can be useful to better understand within-tree water storage (Beyer et al., 2020), an important but often neglected water pool that should be further investigated (Körner, 2019). Over short periods, this pool can be a substantial source for tree transpiration, decoupling water supply and demand (Yan et al., 2020) and thus, increasing drought resilience (Pineda-García et al., 2013; Preisler et al., 2022). Trees store water intracellularly in living cells and in intercellular spaces, and water exchanges between sapwood, inner bark and heartwood tissue (Meinzer et al., 2001; James et al., 2003; Treydte et al., 2021). A clear species-specific relationship exists between plant water potential, VWC_stem_ and hydraulic conductivity (Rosner et al., 2019). With decreasing plant water potential, xylem tension increases, and water is drawn out of increasingly smaller xylem fibers (Hao et al., 2013). If tension gets too high, xylem embolism occurs and within-tree water transport is impaired (Meinzer et al., 2001). So far, time series of stem water content and isotope data have rarely been collected concurrently despite the impacts of internal storage and mixing on the interpretation of xylem water isotope values (Knighton et al., 2020). Furthermore, to the authors' best knowledge, no experiment exists combining tree water storage, sap flow and RWU depths dynamics in tropical systems, despite the close link between the three variables and their resulting interplay in tree drought responses.

To address the above-mentioned research gaps, take advantage of new methodological possibilities and advance our understanding of the role of deep roots in tropical ecosystems, we conducted a drought and re-wetting experiment at Biosphere 2 Tropical Rainforest (TRF). The enclosed model ecosystem offers the possibility to control environmental conditions and access soil from below. After the forest was exposed to a two-month drought, we homogeneously added isotopically labeled water from below to the ecosystem, one week before precipitation resumed. Temporal changes of RWU were observed in ten tree individuals of five different species by monitoring soil water content, sap flow, stem water content and the isotopic composition of soil, tree xylem and transpired water. To capture shifts in RWU depth in adequate temporal resolution, we used in situ methods for all isotopic measurements.

With this experimental setup we (1) determined the percentage of deep-water in RWU during peak drought and following precipitation input and (2) studied how volumetric fluxes of deep and shallow RWU reacted to changing soil moisture availability. By combining all measurements and their temporal changes, we (3) assessed the importance of deep-water access and stem water content for drought resilience of tropical trees.

## 2 Materials and methods

### 2.1 Ambient conditions, soil and selected trees

The experiment was conducted at Biosphere 2 TRF, Arizona, USA within the interdisciplinary B2WALD project (Werner et al., 2021). The 30-year-old mesocosm encloses an area of 1940 m^2^ and contains a variety of plant species from the humid tropics (Rascher et al., 2004) with tree heights up to 25 m. Soils are 0.8–4 m deep and divided into two strata (Scott, 1999). Sandy loam subsoil (64 % sand, 21 % silt, 15 % clay; skeletal content 45 %; bulk density 1.71 g cm^−3^) of low water retention and varying depth is overlaid by 0.8 m loam topsoil (35 % sand, 42 % silt, 23 % clay; skeletal content 22 %; bulk density 1.43 g cm^−3^). At the soil base, perforated PVC drainage pipes (10 cm diameter) prevent water-logging.

**Fig. 1:**
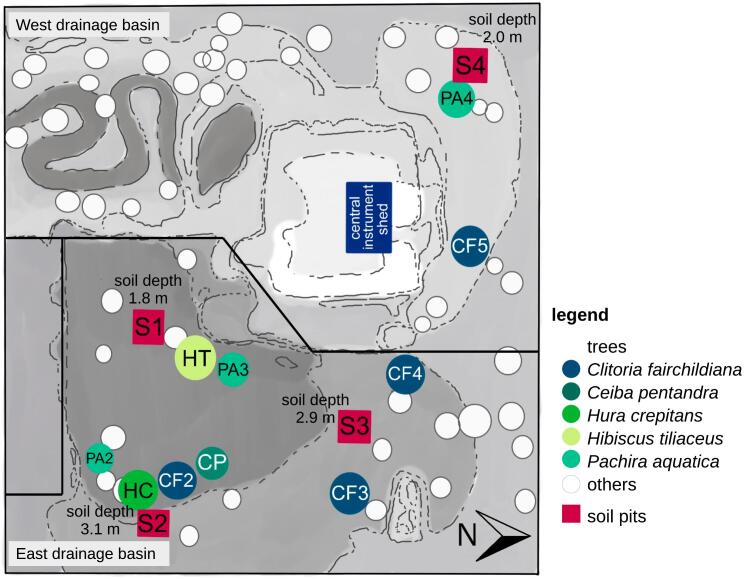
Top view of Biosphere 2 Tropical Rainforest showing the locations of soil pits (S1–4), including their depths, tree individuals and the central instrument shed. Grey shading depicts soil surface elevation with lower areas having darker colors.

We studied ten tree individuals of five different species (Fig. 1, CF: *Clitoria fairchildiana* R.A. Howard, PA: *Pachira aquatica* Aubl., HC: *Hura crepitans* L., CP: *Ceiba pentandra* L., HT: *Hibiscus tiliaceus* L.). For the latter three, only one individual was present. The level of replication was ‘canopy trees’. For CF and PA, we investigated four and three individuals, respectively. Tree height, diameter at breast height (DBH) and soil depth for each of the measured trees are summarized in Table 1.

**Table 1:** Tree height, diameter at breast height (DBH) and soil depth for each of the investigated trees. For tree individuals with multiple stems (a-c), several DBH are reported.

Tree ID	CF2	CF3		CF4			CF5			CP	HC	HT	PA2	PA3	PA4
Stem	–	a	b	a	b	c	a	b	c	–	–	–	–	–	–
DBH [cm]	30.6	27.4	21.0	29.6	17.2	17.0	23.7	22.2	25.5	35.7	46.0	29.3	12.7	12.7	30.1
Height [m]	18.0	20.0		25.0			23.0			20.0	20.5	25.0	8.0	6.0	15.0
Soil depth [m]	2.5	3.3		3.3			1.4			2.5	2.5	2.5	2.5	2.5	1.4

To characterize ambient conditions, we used air relative humidity (RH) and temperature (T_air_) data monitored at 7 m above ground throughout the experiment (Werner et al., 2021).

### 2.2 Irrigation pattern, experimental drought and deep labeling

Under non-drought conditions, rain with constant isotopic composition originating from a local aquifer (δ^18^O = −9.0 ± 0.1 ‰ and δ^2^H = −62.2 ± 0.6 ‰) was provided three times a week by overhead sprinklers, had no seasonality and totaled 1600 mm per year (Werner et al., 2021). The drought started on October 8, 2019 and the first post-drought rain occurred on December 12, 2019 (18 mm). After an additional week without precipitation, we simulated a second precipitation event (19 mm). Regular rain restarted thereafter. For additional details about the controlled ecosystem drought see Werner et al. (2021).

During peak drought (December 2–5, 2019), deep soil water was spatially labeled with a total amount of 23,000 l of water (12 mm) enriched in ^2^H (average δ^2^H ~ 2,300 ‰, δ^18^O = −8.8 ‰). For this, we first closed all drainage outlets. We then pumped labeled water into multiple insertion points distributed across the B2 TRF, i.e., into non-perforated PVC pipes directly connecting the soil surface with the perforated drainage pipes at the soil bottom. To distribute it as homogeneously as possible across the entire base area, we also inserted it at the bases of all four soil pits (Fig. 1). Before this, soil pit walls were additionally covered with plastic foil.

### 2.3 Hydrometric measurements

#### 2.3.1 Soil water content

Soil volumetric water content (VWC_soil_) and matric potential (Ψ_soil_) were recorded across depths in four soil pits (S1–4, Fig. 1) using SMT-100 (Truebner, Neustadt, Germany) and TEROS 21 sensors (Meter Group, Pullman, USA). VWC_soil_ was measured at 5, 10, 20, 50, 100 cm and 200 cm (for S2 and S3 with depths >2 m) and at the soil bottom above the underlying concrete (180 cm, 310 cm, 290 cm and 200 cm for S1-4, respectively). Ψ_soil_ was recorded at 5, 10 and 20 cm depth for all soil pits.

We pooled measurements into three depth compartments (top, middle and deep soil) and calculated averages weighted by soil volume. Topsoil (0–35 cm) featured pronounced reactions to distinct rain events and fast decline of VWC_soil_ at the beginning of the drought. The deep soil (bottom-most 7.5 cm) was impacted by deep labeling. The middle compartment contained the remaining soil in between. Because the three compartments differed in soil volumes, VWC_soil_ is also expressed as total amount of water within a soil column with an area of 1 m^2^. We calculated the amount of unbound water (Ψ_soil_ < −1.5 MPa) within the different compartments. Due to texture differences, water at the same VWC_soil_ is not equally tightly bound across soil depth. For the top strata we used available measurements of Ψ_soil_. For the bottom strata we used literature values for sandy loam (Rawls et al., 1982).

#### 2.3.2 Plant water relations

##### 2.3.2.1 Sap flow

Sap flow sensors (HPV-06, Implexx Sense, Melbourne, Australia) were inserted in trunks of selected tree individuals at 100–130 cm height. As CF individuals had multiple trunks, two sensors were installed in CF3 and CF4 (Werner et al., 2021). Every 15 min, sensors recorded raw data for sap flow calculations and trunk temperatures at two radial depths, 0.5 and 1.5 cm below the bark. After removing outliers and faulty measurements, tree sap flux density (V_h_, cm h^−1^) was calculated from raw measurements by incorporating wood density and stem water content determined from increment cores collected at the beginning of the experiment and correcting for wounding and probe misalignment. Calculations were based on IMPLEXX-SF Software v1 (Implexx Sense, Melbourne, Australia) using the Dual Method Approach to accurately capture both fast and slow flows (Forster, 2019). The calculation method was selected using β = 1 as a threshold (see Forster, 2020). Small data gaps (≤4 h) were filled by linear interpolation. Bigger data gaps (max. five days) during deep labeling were filled by averaging diurnal courses of adjacent days. During times when V_h_ was only available for one of the two sensor positions, linear regressions were used to infer one from the other.

To derive sap flow amounts (l h^−1^) from sap flux density measurements, we estimated sapwood depth for each tree via visual wood color differences of increment cores and by spotting tylosis on scans of increment core slices cut with a microtome. As the transition between sapwood and heartwood was gradual and the different approaches yielded slightly different results, we used a range of possible sapwood depths to account for uncertainty. The minimum sapwood depth was hereby the depth at which actual point measurements were conducted, i.e., 2 cm. We assumed a linear decline of V_h_ from the inner measurement position until the determined sapwood-heartwood boundary.

##### 2.3.2.2 Stem volumetric water content

Tree stem volumetric water content (VWC_stem_) was measured with TDR probes with 10 and 15 cm needle length (TDR, 310H and 315H, Acclima, Meridian, USA) for trees with smaller and bigger DBH, respectively. Prior to insertion of TDR probes, holes were drilled into tree trunks in 1.3 m height with help of a drill-guide (see also Werner et al., 2021). VWC_stem_ was calculated from the apparent dielectric constant using a calibration equation (Constantz and Murphy, 1990). We estimated total stem water volumes before drought from VWC_stem_, DBH and tree height. For this calculation, we used VWC_stem_ determined from destructive increment core samples taken at the beginning of the experiment and multiplied it with tree trunk volumes assuming conical shapes.

##### 2.3.2.3 Leaf water potential

Predawn (Ψ_pd_) and midday (Ψ_md_) leaf water potentials were determined for all tree individuals throughout the experiment using a pressure chamber (Scholander, 1966). For more details on timing and procedure of plant sampling see Werner et al. (2021).

### 2.4 Water stable isotope measurements

Hydrogen and oxygen stable isotope values are reported in δ-notation (Gonfiantini, 1978). Values are reported in per mill (‰) and referenced to the VSMOW-SLAP scale.

#### 2.4.1 Soil

Soil water isotopic composition was determined in situ via direct water vapor equilibration (Rothfuss et al., 2013; Volkmann and Weiler, 2014) in all four soil pits at all depths with VWC_soil_ sensors. Additional probes were installed at 2 cm depth to capture evaporative isotope enrichment throughout the drought. Extensive information on the setup, measurement principle and raw data processing was already detailed by Kübert et al. (2023). In brief, to conduct a measurement, water vapor within a gas permeable probe head at a certain soil depth was drawn into a water isotope analyzer (L2130i, Picarro, Santa Clara, USA) for 20–30 min and averages of δ^2^H, δ^18^O and water vapor mixing ratio (WVMR) were calculated for stable plateaus. From these vapor values and soil temperatures, liquid δ-values were calculated assuming isotopic equilibrium (Majoube, 1971). All sample tubes were heated and flushed regularly to minimize the risk of condensation (Kühnhammer et al., 2022).

For every measurement we calculated a humidity index from measured and theoretical water content at saturation at the respective temperature (Marshall et al., 2020). For depths unaffected by evaporative enrichment and before labeling in case of δ^2^H, we found a linear correlation with measured δ-values. We corrected for this effect, which decreased scatter of δ-values. Due to varying data availability between soil pits, depths and over time, we used smoothing (LOESS regression, span = 0.95) to predict daily δ-values. Soil depth with pronounced jumps in δ-values were split into sections, delineated by time points of water input to account for fast shifts in isotopic compositions. Soil δ-values per depth compartment (compare 2.3.1) were determined from averages weighted by VWC_soil_.

#### 2.4.2 Tree xylem and transpiration

The isotopic composition of transpired water was determined for all measured tree individuals using self-made flow-through leaf chambers integrated into an automated in situ system connected to a water isotope laser (L2120i, Picarro Inc., Santa Clara, USA). For details on materials used, data analysis and calibration procedure we refer to Kübert et al. (2023) and Werner et al. (2021). For this study, daily averages of the transpiration isotopic composition, weighted by the transpiration flux, were used, as those best represent xylem water δ-values (Kübert et al., 2023).

To determine the isotopic composition of tree xylem water in situ, we used the stem borehole equilibration method (Marshall et al., 2020) and a system allowing for automatic switching between xylem water measurement points. For a detailed description and schematics of the in situ setup we refer to Marshall et al. (2020), Beyer et al. (2020) and Kühnhammer et al. (2022). Here, we briefly describe the measurement principle and provide information specific to this experiment.

On August 16, 2019 holes were drilled through the trunks of investigated trees using an increment borer (core diameter 5.15 mm, Haglöf, Långsele, Sweden) in 1.25 m and 0.60 m height for large canopy trees (CF2, CF3, CF4, CF5, CP, HC, HT, PA4) and smaller individuals (PA2, PA3), respectively. In very large individuals, with diameters exceeding the increment corers length, holes were not centered but ran on one side of the tree's trunk. To investigate travel times and mixing of water across tree trunks, additional boreholes were installed in selected trees at the highest point accessible on August 27, 2019. Installation heights were 2.7 m (CF3), 2.25 m (CF4, CP), 5.5 m (CF5), 2.35 m (HC), 2.3 m (HT) and 2.1 m (PA4) above ground. Using a manual increment borer instead of an electric drill has the advantage of decreasing friction and potential evaporation when xylem wood is heated during drilling. Additionally, xylem cores could be sampled and were used to derive wood parameters like water content and wood density, e.g., needed for sap flow calculations. Directly following drilling, holes were rinsed with acetone in the hope it would minimize wound reaction (Marshall et al., 2020). To avoid potential impacts on measured δ-values, we repeatedly flushed boreholes thereafter until the strong acetone smell was no longer perceptible. Nevertheless, both boreholes installed in CP, HC and the top borehole in HT were blocked as consequence of tree wound reaction and could hereafter not be used anymore.

Commercially available Swagelok connectors were used to attach PFA tubing (1/8” OD, Ametek, Nesquehoning, USA) to both sides of each borehole. We increased air tightness around boreholes by fixing the Swagelok connectors to the trees with a combination of different sealing materials (Terostat-II, Kahmann & Ellerbrock, Bielefeld, Germany and Parafilm, Bemis, Wisconsin, USA) placed under stainless steel washers. Those washers were pressed against tree trunks using commercially available lashing straps. All boreholes were combined in one system, that allowed for automatic switching between measurements via solenoid magnetic valves (2-Way Elec. Valve, EC-2-12, Clippard Minimatic, Cincinnati, USA).

To conduct a measurement of one particular borehole, one pair of valves was opened simultaneously (one valve at the inflow and one valve at the outflow) and a dry air stream, regulated by a mass flow controller (FC-260, Tylan General, Eching, Germany), was directed into the borehole at a flow rate of 110 ml min^−1^. Inside the borehole, liquid xylem water evaporated and the vapor equilibrated with it isotopically. Subsequently, sampled water vapor was directed into a water isotopes laser (L2130-i, Picarro, Santa Clara, USA) to determine its isotopic composition and water vapor mixing ratio. One borehole was measured for a time span of 30–60 min. The necessary duration to reach a stable plateau of isotope readings was determined for each borehole separately and depended on tubing length and air tightness. To reduce tubing length, three separate self-made manifolds were distributed across Biosphere 2 TRF. Tubing from manifolds to analyzer was flushed with dry air in between measurements. To decrease the risk of condensation, all sample lines were heated (15 W m^−1^, A. Rak Wärmetechnik, Frankfurt, Germany) and insulated. The amount of air flowing through the system exceeded the amount that is taken in by the isotope laser (approx. 35 ml min^−1^). Excess air was allowed to leak at a split-end close to the analyzer and served to check for sufficient air tightness of the system to avoid atmospheric water vapor intrusion.

From raw data, recorded at a resolution of 1 s, 3-min averages were calculated for stable plateaus at the end of each measurement, before subsequent switching to the next borehole. For instrument calibration, three isotope standards were measured every night (see Kübert et al. (2023) and Werner et al. (2021) for details). Measurements at different WVMR allowed to correct for a device-specific dependency (Schmidt et al., 2010; Kühnhammer et al., 2020). Liquid phase δ-values were calculated from measured vapor values and tree temperatures recorded by sap flow sensors using empirical equations (Majoube, 1971). For borehole δ-values we found correlations to the humidity index (ratio of measured and theoretical WVMR at saturation at the respective temperature, also see Section 2.4.1) and the diagnostic parameter “residuals” provided by the analyzer to detect potential organic contamination. These effects were corrected for with multiple linear regressions.

#### 2.4.3 Estimating deep-water contribution to RWU

We combined collected data, namely soil and transpiration δ^2^H and sap flow amounts, to estimate the amount of soil water taken up from labeled deep soil and overlying soil and observe dynamic changes following precipitation. For this, soil water sources were aggregated into labeled deep soil and unlabeled soil above (top and middle soil combined). Water taken up from the soil is not transpired immediately but lags behind by the time needed for transport through trunks. This time lag can be considerable, especially under drought conditions (Evaristo et al., 2019; Magh et al., 2020; De Deurwaerder et al., 2020). Lag-time ranged between 0 and 10 days. It was determined for every tree by using the first arrival of the tracer signal in transpired water, defined as the time when δ^2^H surpassed the average pre-labeling value plus three standard deviations (sd). We then calculated the daily percentage of deep soil water (U_deep_) in the transpiration stream from the mixture of δ^2^H in the two soil sources (Eq. (1)):

(1) $${\text{U}}_{\text{deep}}=\frac{\delta^{2}H_{\mathrm{transp}}-\delta^{2}H_{\mathrm{topsoil}}}{\delta^{2}H_{\mathrm{deepsoil}}-\delta^{2}H_{\mathrm{topsoil}}}$$

Total water taken up from both soil water sources was calculated by multiplying resulting percentages with sap flow amounts of the corresponding day. Due to uncertainties in sapwood depth estimation, we present results with associated minimum and maximum values.

### 2.5 Statistics

All data analysis and visualization were conducted in R (R Core Team, 2022). To compare measurements across the experimental period, we defined sections each spanning one week of data (see Table 2). Average values per section are presented with their associated sd. Percentage changes between experimental sections were calculated for daytime (7:00–17:00) sap flux density and VWC_stem_, and their statistical significance was assessed with the non-parametric Mann-Whitney-U-Test.

**Table 2:** Beginning and end dates of sections each spanning one week of data to compare sap flux density and stem water content measurements between different phases of the experiment.

Section name	Section nr.	Start date (incl)	End date (incl)
Pre-drought	1	30.09.2019	06.10.2019
Peak drought	2	24.11.2019	30.11.2019
Deep rewet	3	05.12.2019	11.12.2019
First rain	4	12.12.2019	18.12.2019
After 2–3 weeks with regular rain	5	02.01.2020	08.01.2020

## 3 Results

### 3.1 Environmental conditions and soil water

**Fig. 2:**
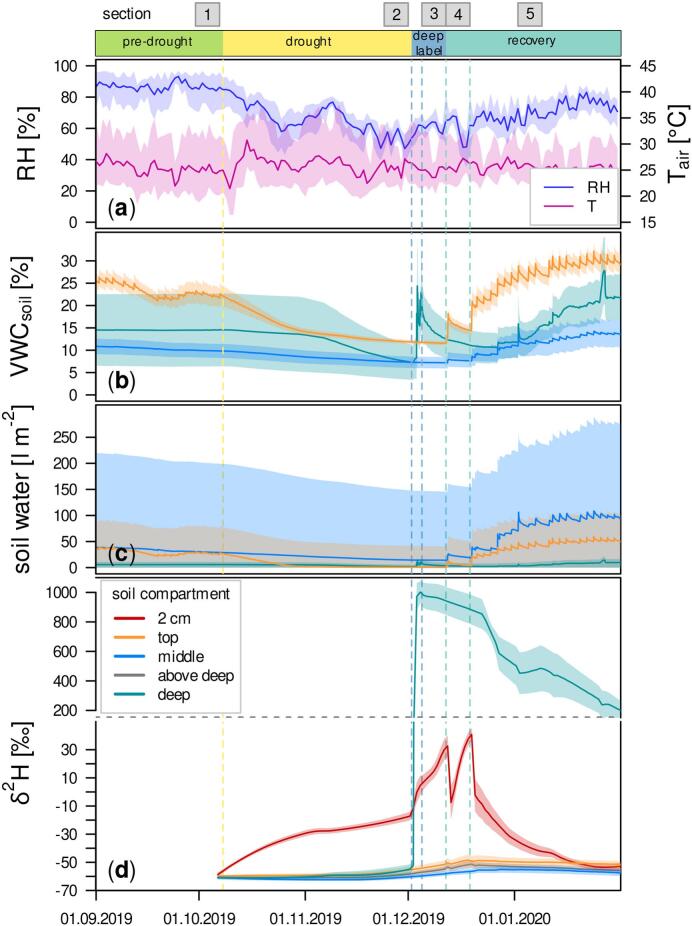
Time courses of ambient air temperature (T_air_) and relative humidity (RH) are shown in panel a. Panel b and c depict soil water status in different depth compartments (top: 0–35 cm, deep: bottommost 7.5 cm, middle: everything in between), expressed as volumetric water content (VWC_soil_) and total (filled area) and unbound water amount (Ψ_soil_ < −1.5 MPa, lines) in an exemplary soil column with 1 m^2^ surface area. The corresponding soil water δ^2^H values are drawn in panel d, an additional isotope probe was installed in 2 cm depth to capture evaporative enrichment at the soil surface. For T_air_ and RH the polygons enclose daily minima and maxima, soil water data is reported as daily averages of all sensor data with their associated standard errors. Vertical dashed lines indicate beginning of drought (yellow), first and last day of deep labeling (dark blue) and first and second post-drought rain (light blue).

The drought and water additions from the bottom and the top shaped soil water availability and atmospheric demand throughout the experiment (Fig. 2). While T_air_ did not change systematically with drought, RH started decreasing immediately after the last rain event, was significantly lower at peak drought and, compared to pre-drought values, remained decreased after the onset of regular rain (Fig. 2a).

We observed different responses to drought and re-wetting among the soil compartments investigated. VWC_soil_ in the topsoil decreased markedly within the first three weeks of drought (Fig. 2b). The reduced decline in VWC_soil_ thereafter coincided with the time period for which topsoil water was below −1.5 MPa, i.e., less available for plants (Fig. 2c). In the middle soil compartment, VWC_soil_ was overall lower than in the topsoil and its decline less steep and more constant over time (Fig. 2b). In contrast to the topsoil, unbound water (>−1.5 MPa) within this compartment was available throughout the experiment (Fig. 2c). Deep VWC_soil_ remained constant until three weeks into the drought (Fig. 2b). The decline afterwards was mainly caused by one soil sensor (S3), which had a higher pre-drought VWC_soil_ (38.2 ± 0.0 %) compared to the other soil pits (6.6 ± 4.0 %). Deep labeling sharply increased VWC_soil_ in deep soil, followed by a rapid, exponential decline. The first rain increased VWC_soil_ in the near-surface soil. The entire soil profile was only re-wetted following repeated rain events.

δ^2^H in the top and middle soil increased until the first post-drought rain event. ^2^H enrichment was most pronounced close to the surface and its magnitude decreased with soil depth (Fig. 2d). Throughout the drought, δ^2^H values at 2 cm depth coincided with the decrease of VWC_soil_ as a consequence of soil evaporation. Deep soil water δ^2^H values strongly increased following label application. Concurrently, we observed an increase in soil δ^2^H in 2 cm depth that coincided with ^2^H enrichment of atmospheric vapor (data not shown) and was not accompanied by an increase in VWC_soil_. The ^2^H enrichment of shallow soil water was thus likely caused by ambient air intrusion (see also Rothfuss et al., 2015; Kühnhammer et al., 2020).

δ^18^O values in middle and deep soil water stayed constant (−8.9 ± 0.3 ‰), reflecting rain water (data not shown). ^18^O enrichment was strongest at 2 cm depth and also visible in the topsoil. The highest δ-values were measured right before rain re-wetting started and were −0.4 ‰ and −7.6 ‰, respectively.

### 3.2 Tree water status, transport, storage and δ^2^H

**Fig. 3:**
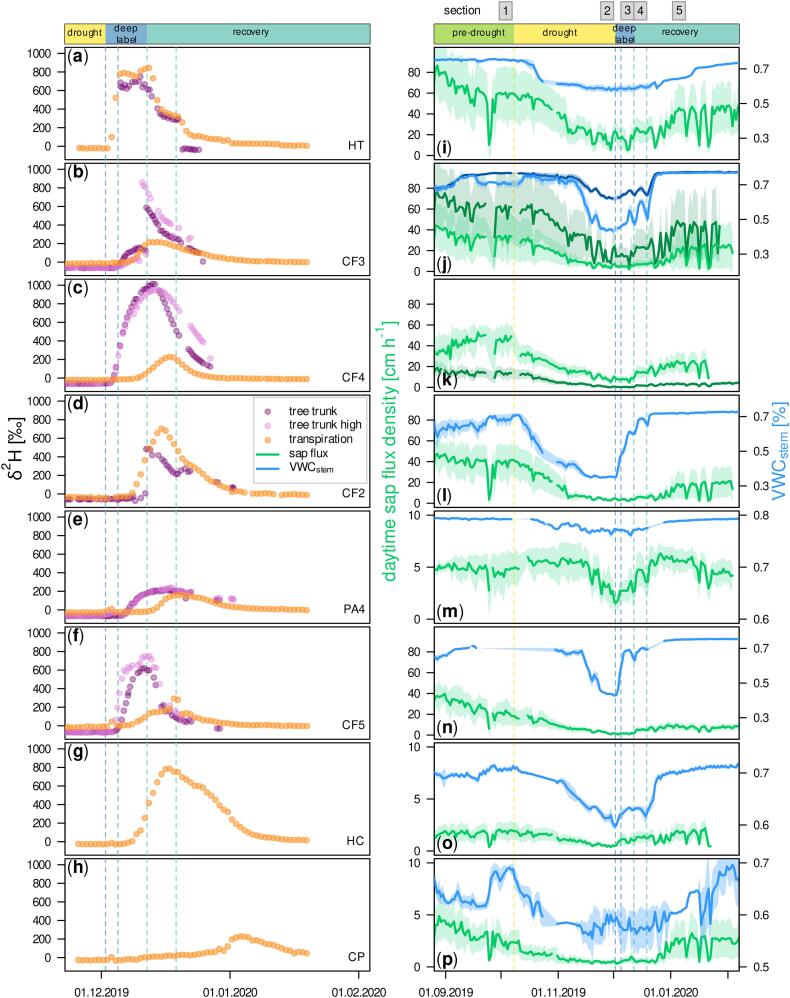
Time series of daytime (7:00 to 17:00) sap flux density is displayed along with stem water content (VWC_stem_, panel i-p) and δ^2^H of transported water (panel a-h) in the different tree compartments monitored. Plots were arranged according to average daytime sap flux density in the time period from deep labeling until the onset of regular precipitation with HT (on top) and CP (at bottom) featuring the fastest and slowest sap flow, respectively. Vertical dashed lines indicate beginning of drought (yellow), first and last day of deep labeling (dark blue) and first and second post-drought rain (light blue).

Tree sap flow and water storage changed dynamically with soil water availability and ambient conditions (Fig. 3i-p). Deep labeling increased δ^2^H in transpiration and xylem water for all canopy trees (Fig. 3a-h). Only the two small understory trees (PA2, PA3) did not show pronounced uptake of labeled deep-water (data not shown). We will hence focus on canopy trees with access to deep labeled soil water.

All trees showed a significant reduction (average: −76.0 ± 18.1 %) of daytime sap flux density when comparing pre-drought values with the last week of the drought (Table 3). Following deep-water addition, two trees (HC and CP) increased their sap flux density. For all other tree individuals, sap flux densities increased again with the first rain and even more strongly with the onset of regular rain events. VWC_stem_ decreased with increasing drought conditions. Timing, extent and velocity of this decrease differed among measured trees (Fig. 3i-p). In contrast to sap flux density, VWC_stem_ recovered with deep-water addition for all CF individuals and HC. After the restart of regular rain, all trees again had higher VWC_stem_ than during peak drought.

**Table 3:** Percentage changes of daytime sap flux density (7:00 to 17:00) and stem water content (VWC_stem_) between sections and their statistical significance. Also shown is Ψ_pd_ for selected experimental dates. Significance levels: *** *p* < 0.001, ** *p* < 0.01, * *p* < 0.05, ns not significant.

			HT		CF3		CF4		CF2		PA4		CF5		HC		CP	
Section comparison																		
Sap flux density change [%]	Drought reduction	1:2	−74.7	***	−80.2	***	−86.0	***	−91.4	***	−30.7	***	−89.7	***	−74.4	***	−80.6	***
	Change with deep label	2:3	−6.1	ns	2.2	ns	−5.5	ns	−2.9	ns	−2.3	ns	−26.2	ns	107.0	***	57.2	**
	Change with first rain	2:4	59.2	***	58.6	**	86.4	***	58.3	**	37.2	*	133.4	**	161.1	***	27.5	**
	Change with regular rain	2:5	186.4	***	242.7	***	218.3	***	373.6	***	79.0	***	295.3	***	263.8	***	483.3	***
VWC_stem_ change [%]	Drought reduction	1:2	−22.3	***	−27.4	***	–		−48.6	***	−2.9	**	−37.9	**	−13.1	***	−14.1	***
	Change with deep label	2:3	0.3	ns	7.5	***	–		60.1	***	−0.2	ns	51.5	***	2.3	**	−1.7	ns
	Change with first rain	2:4	1.7	**	13.6	***	–		89.0	***	0.4	ns	55.0	***	2.2	**	−2.3	*
	Change with regular rain	2:5	10.4	***	44.0	***	–		105.0	***	2.5	***	70.3	***	14.2	***	3.6	***

Section																		
Ψ_pd_ [MPa]	Pre-drought	1	−0.5		−0.1		−0.1		−0.1		−0.1		−0.1		−0.1		−0.5	
	24.11.2019	2	−1.6		−1.0		−0.9		−0.8		−0.6		−1.5		−0.8		−0.5	
	06.12.2019	3	−1.1		−0.4		−0.6		−0.7		−0.3		−0.5		−0.6		−0.4	
	13.12.2019	4	−0.7		−0.4		−0.8		−0.4		−0.3		−0.6		−0.4		−0.4	
	20.12.2019	5	−0.6		−0.4		−0.5		−0.4		−0.3		−0.3		−0.4		−0.5	

Ψ_pd_ (Table 3) decreased with drought for all trees and was particularly low at peak drought for HT and CF5. Values increased following deep-water addition and re-wetting from the top but were still below pre-drought values after the second rain event. Values for Ψ_md_ were between −0.1 MPa (CP) and − 0.6 MPa (CF4) before the onset of the drought. At peak drought we found values below −1.0 MPa for all tree individuals, except HC and CP (−0.7 and − 0.3 MPa, respectively). The lowest value (−2.4 MPa) was determined for HT. For timelines of Ψ_pd_ and Ψ_md_ see Fig. A1.

Average isotopic composition measured in tree trunks was close to precipitation water before label addition with −10.0 ± 1.3 ‰ and −61.4 ± 7.5 ‰ for δ^18^O and δ^2^H, respectively. Daily δ-values measured in transpiration were similar for δ^18^O (−9.8 ± 3.0 ‰) but higher for δ^2^H (−28.6 ± 12.0 ‰). δ^18^O measured in tree trunks and transpiration did not change notably throughout the experiment. The deep-water label increased δ^2^H in all measured canopy trees (Fig. 3a-h). However, the time course, velocity and magnitude of δ^2^H increase differed between tree individuals. For instance, HT featured step-wise changes of δ^2^H in reaction to deep labeling and subsequent input of unlabeled precipitation (Fig. 3a). In contrast, all other trees showed a more gradual change of δ^2^H and some showed a temporal delay of the label signal from trunks to leaf transpiration.

CF individuals featured a pronounced difference in xylem water δ^2^H not only when comparing xylem and transpiration measurements but also depending on the direction of the borehole air stream, i.e., borehole sample side. Sharp increases in xylem δ^2^H of CF3 and CF2 (compare Fig. 3b and d) are associated with times when borehole side connections were changed.

### 3.3 Contribution of deep soil water to tree root water uptake

**Fig. 4:**
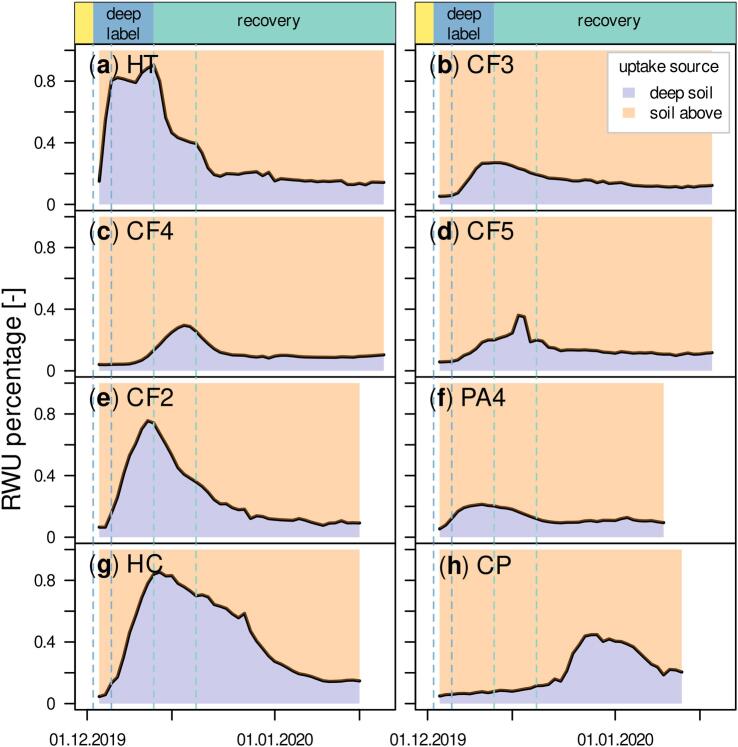
Contribution of labeled deep soil water and water from overlying soil to transpired water of investigated trees. The temporal delay of label signal arrival varied between individuals and was up to 10 days in case of PA4. For presented uptake percentage plots we moved transpiration time series backwards in order to represent temporal dynamics at the location of root water uptake. Vertical dashed lines indicate first and last day of deep labeling (dark blue) and first and second post-drought rain (light blue).

The highest (90 %) and lowest (21 %) maximum contribution of deep-water in transpiration was found for HT and PA4, respectively and temporal contribution dynamics also differed between tree individuals (Fig. 4). For HT (Fig. 4a), deep soil water percentage decreased rapidly from December 13, 2019, right after the first rain event while CP (Fig. 4h) only reached maximum δ^2^H on December 29, 2019, which is 17 days after the first rain event. All trees still transpired water with elevated δ^2^H at the end of isotope measurements on January 19, 2020, after one month with regular rain. Deep-water contributions by then ranged between 9 and 20 % for CF2 and CP, respectively.

**Fig. 5:**
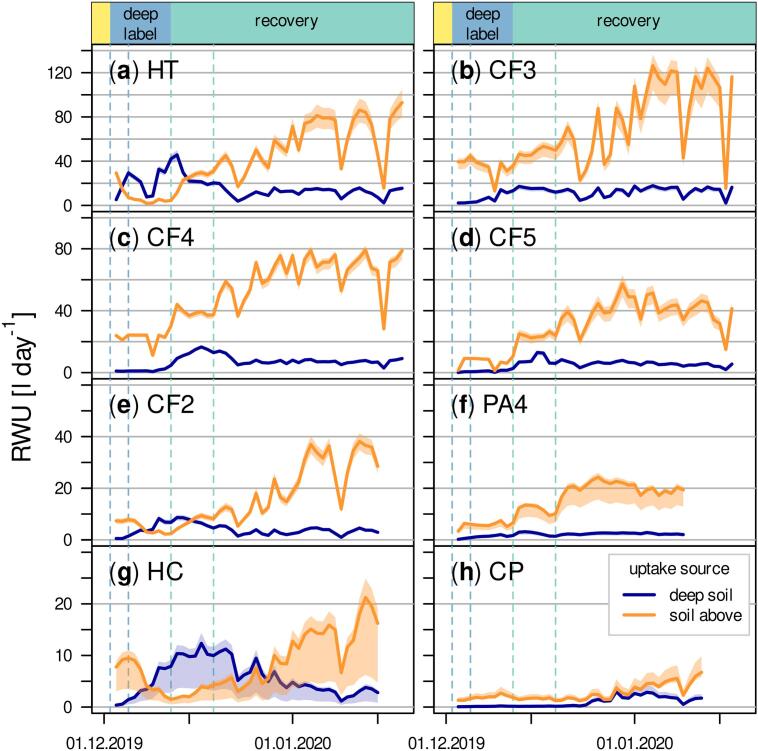
Total daily water amounts taken up from deep-water and soil water above calculated from source water shares and cumulated daily sap flow amounts for each tree individual. Vertical dashed lines indicate first and last day of deep labeling (dark blue) and first and second post-drought rain (light blue).

With 45.7 l day^−1^, the highest volumetric water uptake from deep soil was found for HT (Fig. 5a). The highest uptake from topsoil was recorded for CF3 and amounted to 126.3 l day^−1^ (Fig. 5b). This indicates that volumetric uptake from deep soil was limited in quantity. Generally, water taken up from unlabeled soil increased substantially and quickly following the first rain event for tree individuals with higher sap flux densities, namely HT, CF3, CF4 and CF5. It was also more variable compared to RWU from deep soil.

## 4 Discussion

We investigated the contribution of deep-water to RWU including its dynamics with changing ambient conditions, and assessed how this deep-water source might help mitigate impacts of drought. TRF provided the unique possibility to homogeneously label deep soil water which, to the authors best knowledge, was not attempted previously. We combined deep labeling with water content, water flux and in situ water stable isotope measurements in soils and plants. This enabled us to quantify the specific contribution of deep soil to plant transpiration not only with re-wetting after drought (e.g., Evaristo et al., 2019) but during the time of lowest surface VWC_soil_ and also observe dynamic changes in RWU depth.

### 4.1 Deep-water uptake

The deep soil contribution of 21 % - 90 % to total RWU was remarkable, considering it was applied as a pulse with limited quantity and only affected <5 % of total soil volume. It was notable that all canopy trees, independent of species, had access to deep-water, located at depth between 1.4 and 3.3 m, suggesting that deep roots are common also in environments without seasonally recurring dry periods.

Supporting these high deep-water uptake percentages, studies interpreting fluctuations of VWC_soil_ across depths found that deep roots contributed substantially to RWU and uptake percentages increased with limited surface soil water availability during the dry season (Bruno et al., 2006; Christina et al., 2017), especially under rainfall exclusion (Markewitz et al., 2010; Davidson et al., 2011). All mentioned studies concluded that RWU occurred at soil depth > 10 m and uptake percentages were within the range found in our experiment. Highest dry season RWU percentages of 72.8 % from soil depth >1 m (Christina et al., 2017) and 72 % from soil depth >2 m (Bruno et al., 2006) were reported. Results from studies applying the isotopic method were less uniform. While Stahl et al. (2013) and Brum et al. (2019) concluded that about half of the investigated tropical trees mainly extracted soil water below 1 m soil depth during the dry season, Romero-Saltos et al. (2005) did not find any RWU from depth below 1.5–2 m even in the dry season under rainfall exclusion. Similarly, Sohel et al. (2021) found a dominant contribution (75 %) of water from the top 20 cm. While discrepancies in prevalence and importance of deep roots could originate from different tree species, landscape positions and ecosystems studied, it could also arise from limited temporal resolution of obtained isotope data.

Even though deep-water made up a large maximum proportion of RWU, its quantity was limited. Particularly for trees with high sap flow rates, the water amount taken up over the drought followed the course of surface soil water depletion and only increased again with precipitation input (Fig. 3i-p). This interdependence was caused by increased stomatal control and leaf shedding, which are previously reported drought responses for TRF trees (Rascher et al., 2004) and greatly affected carbon sequestration (Rascher et al., 2004; Werner et al., 2021). Deep roots did not fully compensate for reduced uptake from surface soil, despite their potentially larger vessels (Wang et al., 2022) and more vessel-to-vessel connections (Johnson et al., 2014), leading to higher hydraulic conductivity. For some trees, in particular HT, CF4 and HC, we calculated higher total uptake amounts from deep-water before precipitation restarted, while the other investigated tree individuals showed rather constant deep RWU amounts (compare Fig. 5). Similarly, Gessler et al. (2022) reported that beech trees did not quantitatively increase RWU from the deepest soil layer, in their case from 45 cm soil depth, under drought conditions. Deep-water uptake was likely restricted by a low root length density, that typically decreases exponentially with soil depth (Schenk and Jackson, 2002; Davidson et al., 2011; Moser et al., 2014). This is also the case for TRF, where roots were primarily found within the upper 65 cm with a share of 60 % in the top 15–20 cm (Evaristo et al., 2019).

### 4.2 Restricted RWU from unlabeled upper soil

The high deep-water uptake percentages observed in the B2WALD drought demonstrate that RWU from overlying soil was strongly limited, even though unbound water (Ψ_soil_ > −1.5 MPa) was still available in the middle soil compartment. This could indicate that few fine roots were present therein and/or the measured trees developed higher root length densities above the impermeable concrete floor as observed in a column experiment (Deseano Diaz et al., 2022). This deep root growth might be a response to the current or previous droughts (Fan et al., 2017), and could be the reason for the observed mid-drought decrease in deep-water, thus indicating the below-ground drought adaptability of trees in the humid tropics. Possible explanations are also a depletion of water around roots in the upper soil compartments, especially if soil hydraulic conductivity was not high enough to replace water taken up previously (Javaux et al., 2008). Shrinking could also have disconnected roots from surrounding soil matrix (Carminati et al., 2009). Ψ_pd_ represents Ψ_soil_ at the location of active RWU (Améglio et al., 1999; Stahl et al., 2013). At peak drought measurements were above −1 MPa for all individuals but HT (−1.6 MPa) and CF5 (−1.5 MPa), supporting restricted RWU from dry topsoil. It could be beneficial for tree individuals and entire ecosystems, if trees reduce RWU, e.g., through stomatal regulations, to prolong drying of the soil tapped by roots and hence decrease the risk of hydraulic failure.

### 4.3 Dynamic shifts in RWU depth

The rapid increase in RWU following precipitation highlights the quantitative importance of precipitation and surface-near soil water. To make most use of incoming precipitation, trees must ensure that roots within the topsoil stay alive throughout the drought, which might be a reason why all canopy trees accessed deep-water. This can potentially be facilitated by hydraulic lift, transporting water from deep to shallow soil layers (Oliveira et al., 2005). However, if this played a role, trees only transported water in limited quantity to their own rhizosphere, as we did not see any corresponding increase of VWC_soil_ and the observed increase of soil δ^2^H in 2 cm depth matched ambient air δ^2^H.

Fast changes, i.e., within a few days, in RWU depth were also observed for a number of tropical trees by Stahl et al. (2013). These shifts were attributed to fluctuations of VWC_soil_ across soil depth. Globally, Miguez-Macho and Fan (2021) found that 70 % of plant transpiration is sourced from current precipitation but highlighted the plasticity of RWU, with deep-water contributing 83 % in winter-dry tropics during the driest month. In contrast, Borma et al. (2022) found a strong reliance of the studied tropical evergreen forest on recent precipitation during both wet and dry season and concluded that this challenges the role of deep roots as a central drought-mitigation mechanism.

In light of our experiment, those observations and contrasting conclusions drawn from isotope studies (see Section 4.1) are not necessarily contradictory nor do they reject the occurrence nor the importance of deep roots. Instead, they could be an artifact of highly-dynamic changes that could hardly be captured with destructive sampling approaches. The certainty that we do not miss any temporal changes in soil and xylem water, that would greatly impact calculations of RWU depths, emphasizes the value of temporally high-resolved in situ isotope data. While our results confirm that precipitation and surface soil water is quantitatively the dominant water source, we found deep-water access to be a species-independent trait, providing an essential water source for canopy trees during reduced precipitation input. Consistent with these findings, Miller et al. (2010) found that deep roots with groundwater access were critical for tree survival during dry periods in Californian blue oaks but that uptake was insufficient for trees to thrive.

### 4.4 Impact of deep-water access on plant water status

Despite the lack of consistent increase in sap flow and hence transpiration following deep-water addition, the mitigating impact of deep roots on plant water stress became evident with the increase in Ψ_pd_ that occurred quickly after deep-water addition for all measured tree individuals. Without deep-water access, trees would likely have further decreased transpiration rates or leaf water potentials with possibly fatal consequences arising from increased xylem embolism in hydraulically-vulnerable tropical trees (Chitra-Tarak et al., 2021). Considering biomass share and cumulative yearly uptake amounts of the large canopy and emergent trees, deep roots are disproportionally relevant to endure drought periods and maintain basic tree functioning, as recently highlighted at the global scale (Miguez-Macho and Fan, 2021).

### 4.5 Connection of VWC_stem_ to deep-water access and drought resilience

All measured canopy trees had access to deep-water. However, water uptake amount, drought and re-wetting responses differed for every tree individual investigated. Particularly striking were differences in the plasticity and temporal dynamics of VWC_stem_. This is especially interesting, considering the above-mentioned uniform increase in Ψ_pd_ with deep-water addition and emphasizes the need to further investigate in future experiments how tree water status, use and content mutually affect each other.

Our data support the importance of VWC_stem_ as buffer for decreased RWU on diurnal and to greater extents across longer timescales, i.e., seasons or drought periods (Meinzer et al., 2008; Pineda-García et al., 2013; Yan et al., 2020). Estimated total stem water volumes ranged between 0.26 m^3^ (CF2) and 0.62 m^3^ (CF5), which, assuming this water is the only source, supply three and five days of pre-drought sap flow amounts, respectively. However, for CP, the tree with the lowest daily sap flow amounts, stored water (0.39 m^3^) would last for 60 days. To maintain its function as a buffer that supports high transpiration rates under conditions of delayed replenishment from soil water, water withdrawn from stem water storage is refilled during the night (Oliva Carrasco et al., 2015). In our experiment, the fast decline in VWC_stem_ observed for most species during the drought suggests that the stem water reservoir was quickly exhausted when soil water supply was restricted even though reduced sap flow decreased daily withdrawal from stem water storage. The decline coincided with points in time when trees could not refill VWC_stem_ during the night anymore, and values only increased again after additional water supply. Similarly, Yan et al. (2020) found a connection between seasonal soil water availability and stem water content when modeling stem water dynamics of tropical tree species. The authors point out that model validation was limited due to missing field observations of stem-water dynamics, which emphasizes the value of the collected dataset to improve physically-based ecohydrological modeling.

Notably, all CF individuals showed an immediate and steep increase of VWC_stem_ with addition of deep-water. To smaller extent this was also observed for HC. This indicates that, especially drought-sensitive CF individuals with high pre-drought sap flow rates, use stem water storage flexibly. A similar link between evaporative demand and high stem water capacitance, facilitating large and fast withdrawal and refilling of VWC_stem_, was observed by Oliva Carrasco et al. (2015). Deep-water refilled xylem vessels and likely was used for embolism repair. The extent and functioning of repair is however still controversial (Klein et al., 2018). Decoupling of transpiration from soil water via VWC_stem_ also provides an explanation for the long traceability of label water in transpired water as also concluded by James et al. (2003).

The interruption of radial water flow, expressed by diurnal VWC_stem_ fluctuations, and cessation of sap flow were identified as indicators for irreversible tree damage leading to mortality (Preisler et al., 2021). Even though trees, especially those with high maximum sap flux densities, showed pronounced changes in their hydraulic functioning in response to drought and strongly decreased their sap flow to values close to zero, they recovered with addition of deep-water and even more with the first rain events, showing a surprising flexibility to the drought conditions they were exposed to. This observation coincides with the commentary by Körner (2019) that states that hydraulic failure of water transporting conduits is not the cause for tree mortality but rather a symptom of drought stress and that the relation of VWC_stem_ decrease, xylem embolism and fatal dehydration of essential tissues is highly species-specific.

### 4.6 Sectorial water transport from deep roots and other study limitations

An in-depth look at isotope data from different compartments of single trees, i.e., trunk xylem in different heights and transpiration, revealed pronounced differences in δ^2^H temporal dynamics and maximum values for some individuals investigated. These differences cannot be explained by temporal delay due to sap flow transport time nor by increasing mixing with storage water. Rather, they indicate that water taken up by deep roots was not well-mixed within tree xylem. A high degree of sectoriality, i.e., restricted cross-sectional mixing of water and nutrients (Ellmore et al., 2006), was previously observed for lateral roots (Nadezhdina, 2010; Volkmann et al., 2016) but has not yet been observed for deep roots. It could be important to reduce the spread of embolism in tropical diffuse-porous species with large and highly conductive vessels (Zanne et al., 2006). The high heterogeneity for CF individuals could arise from their strong decline in VWC_stem_ pointing towards loss of cross-sectional hydraulic conductivity (Rosner et al., 2019). This surely complicated our deep-water uptake calculations and should be further investigated and considered in future research looking at the contribution of deep roots to RWU, especially under conditions of limited soil water availability.

The possibility of incomplete mixing of water within tree sapwood could potentially propagate across tree height and cause heterogeneity within tree crowns. While fixed measurement points and higher temporal resolution of in situ isotope methods enable us to investigate temporal dynamics of water uptake patterns, the representation of spatial heterogeneity remains limited (Beyer et al., 2020). This could be tackled in future studies by installing multiple boreholes and leaf chambers in one tree individual in different orientations and heights.

While the ability to control ambient conditions and to access deep soil at B2 TRF was an indispensable prerequisite for the presented experiment, the fully enclosed ecosystem features some differences to natural systems: The glass housing reduces incoming solar radiation and creates a marked vertical temperature gradient with limited turbulent mixing in and above the canopy (Arain et al., 2000). The concrete bottom restricts rooting depth. Furthermore, the selection of tree individuals is constrained and only one fully-grown individual was present for all species but CF, which prevented us from drawing conclusions on species-specific RWU strategies. Lastly, we introduced labeled water to deep soil during a severe drought, when below-ground water availability was strongly reduced. The importance and temporal dynamics of water uptake from deep roots should be further investigated under different preconditions in future experiments.

## 5 Conclusion

By combining spatial labeling of deep soil water with in situ isotope methods and quantitative measurements of water fluxes and water content at the soil-plant-interface, we observed deep RWU and followed its path through the trees into the atmosphere. Our results suggest that deep roots (>1 m) are a common feature among tropical trees even without exposure to seasonal dry periods. During times of limited surface soil water availability, deep soil provided an essential water source, especially for trees with high sap flow rates. With access to deep-water, plants could maintain higher water potentials and replenish their stem water content, potentially reducing the extent of xylem embolism and consequential die-off. Nevertheless, deep RWU was limited in quantity and could not compensate for reduced uptake from the topsoil. Therefore, cumulative water fluxes of this tropical ecosystem were driven by precipitation input. Our results confirm that tropical trees switch their water uptake depth depending on soil water status not only seasonally but dynamically within as little as a few hours in response to rainfall. The wealth of data collected in B2WALD provides an exceptional opportunity to track water movement across a well-constrained artificial ecosystem, improve isotope-enabled models and advance process-representation in land-surface models.

The following is the supplementary data related to this article.

Fig. A1 Timelines of predawn (Ψ_pd_) and midday leaf water potential (Ψ_md_) for investigated tree individuals. Darker points are mean values, calculated from single measurements per timepoint, depicted by lighter colors. Vertical dashed lines indicate first and last day of deep labeling (dark blue) and first and second post-drought rain (light blue). Fig. A1

## Funding

Our work was supported by the 10.13039/501100001663Volkswagen Foundation (contract no. A122505; reference no. 92889 to MB), the 10.13039/100010663European Research Council (ERC consolidator grant #647008 VOCO2 to CW) and the Philecology Foundation to LKM.

## Author contribution

MB, MD, JvH, CW, NL, LKM, AK and KK designed the experiment. It was mainly conducted by KK, AK, KB and JvH. Data analysis was led by KK and AK with input from all authors. All authors contributed to the interpretation of obtained results. KK wrote the manuscript with input from MB, CW, MD and NL. All authors revised the manuscript.

## Declaration of competing interest

The authors declare that they have no known competing financial interests or personal relationships that could have appeared to influence the work reported in this paper.

## Data Availability

The data that support the findings of this study are available from the corresponding author upon reasonable request.
