# Supplementary material for: Deep roots mitigate drought impacts on tropical trees despite limited quantitative contribution to transpiration
Source: Sci Total Environ. 2023 Oct 1;893:164763. doi: 10.1016/j.scitotenv.2023.164763 (PMC10331952; doi:10.1016/j.scitotenv.2023.164763)
Supplement: Fig. A1 — Timelines of predawn (Ψpd) and midday leaf water potential (Ψmd) for investigated tree individuals. Darker points are mean values, calculated from single measurements per timepoint, depicted by lighter colors. Vertical dashed lines indicate first and last day of deep labeling (dark blue) and first and second post-drought rain (light blue). [file mmc1.pdf]

Supplementary material

Deep roots mitigate drought impacts on tropical trees despite limited quantitative contribution to transpiration

Kathrin Kühnhammer[1,2], Joost van Haren[3,4], Angelika Kübert[2,5], Kinzie Bailey[6], Maren Dubbert[2,7], Jia Hu[6], S. Nemiah Ladd[2,8], Laura K. Meredith[3,6], Christiane Werner[2], Matthias Beyer[1]

[1] IGOE, Environmental Geochemistry, TU Braunschweig, Langer Kamp 19c, 38106 Braunschweig, Germany

[2] Ecosystem Physiology, University of Freiburg, Georges-Köhler-Allee 53/54, 79110 Freiburg, Germany

[3] Biosphere 2, University of Arizona, 32540 S Biosphere Road, Oracle, AZ 85623, USA

[4] Honors College, University of Arizona, 1101 E. Mabel St., Tucson, AZ 85719, USA

[5] Institute for Atmospheric and Earth System Research, University of Helsinki, P.O. Box 68 (Pietari Kalmin katu 5), 00014 Helsinki, Finland

[6] School of Natural Resources and the Environment, University of Arizona, 1064 E Lowell St, Tucson, AZ 85721, USA

[7] Isotope Biogeochemistry and Gasfluxes, ZALF, Eberswalder Straße 84, 15374 Müncheberg, Germany

[8] Department of Environmental Sciences, University of Basel, Bernoullistrasse 32, 4056 Basel, Switzerland

Corresponding author: Kathrin Kühnhammer

Technische Universität Braunschweig
Institut für Geoökologie
Langer Kamp 19c
38106 Braunschweig
Germany

Mail: k.kuehnhammer@tu-braunschweig.de
Tel: +49 (0)761 203 8308

Supplementary Figure A1

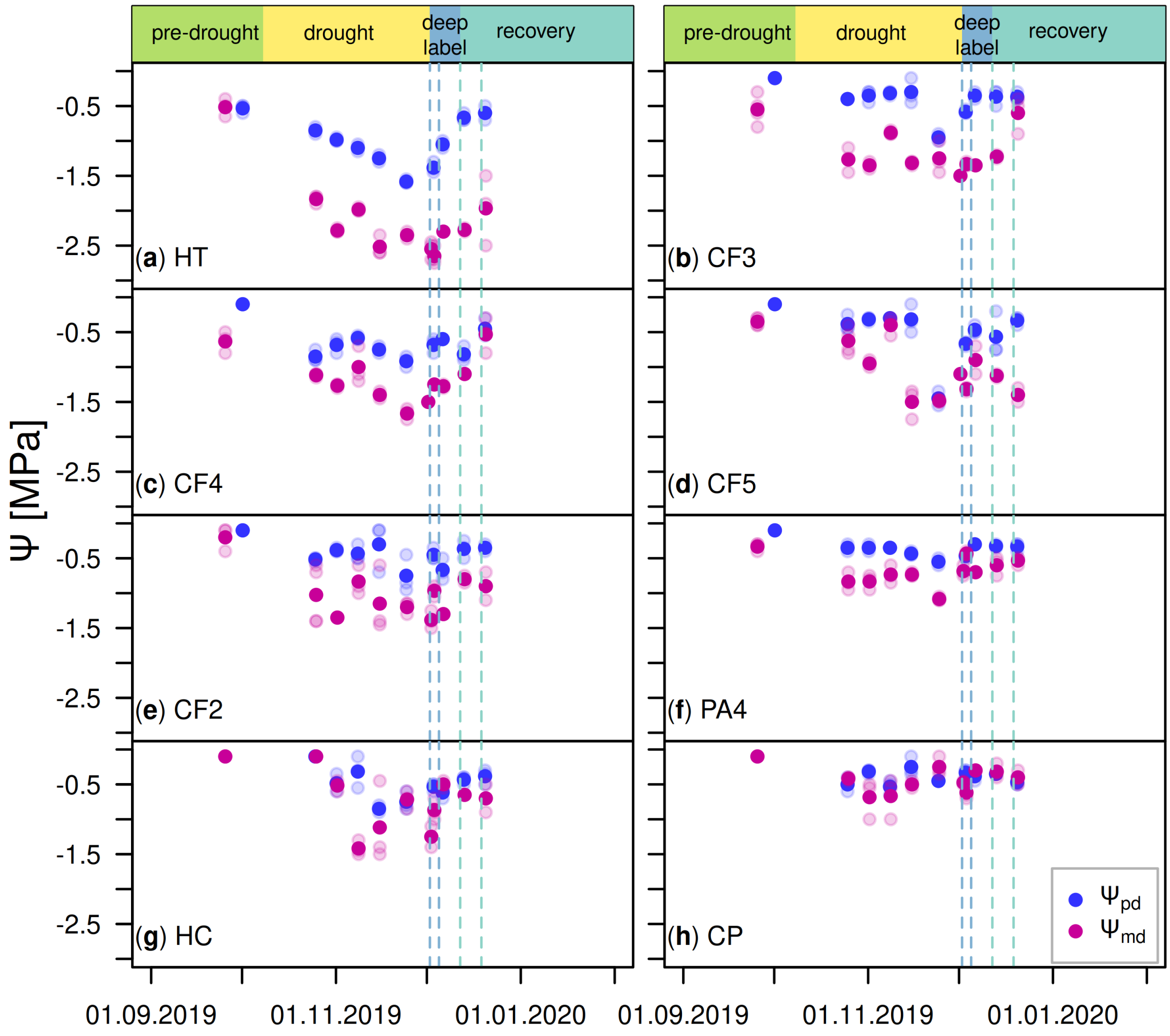

Figure A1: Timelines of predawn (Ψ_{pd}) and midday leaf water potential (Ψ_{md}) for investigated tree individuals. Darker points are mean values, calculated from single measurements per timepoint, depicted by lighter colors. Vertical dashed lines indicate first and last day of deep labeling (dark blue) and first and second post-drought rain (light blue).
